# “I just wanted to speak to someone- and there was no one…”: using Burden of Treatment Theory to understand the impact of a novel ATMP on early recipients

**DOI:** 10.1186/s13023-023-02680-y

**Published:** 2023-04-17

**Authors:** Ian Litchfield, Melanie J. Calvert, Francesca Kinsella, Nisha Sungum, Olalekan L. Aiyegbusi

**Affiliations:** 1 Institute of Applied Health Research, University of Birmingham, Edgbaston, Birmingham, B15 2TT UK; 2 Centre for Patient Reported Outcomes Research, University of Birmingham, Birmingham, UK; 3 NIHR Birmingham Biomedical Research Centre, University of Birmingham, Birmingham, UK; 4 NIHR Blood and Transplant Research Unit (BTRU) in Precision Transplant and Cellular Therapeutics, University of Birmingham, Birmingham, UK; 5 Applied Research Collaboration (ARC) – West Midlands, Birmingham, UK; 6 Present Address: Institute of Immunology and Immunotherapy, University of Birmingham, Birmingham, UK; 7 Birmingham Health Partners (BHP) Centre for Regulatory Science and Innovation, University of Birmingham, Birmingham, UK; 8 Centre for Clinical Haematology, University Hospitals Birmingham NHS Foundation Trust, Birmingham, UK; 9 Midlands and Wales Advanced Therapy Treatment Centre, University Hospitals Birmingham NHS Foundation Trust, Birmingham, UK; 10 Research Development and Innovation, University Hospitals Birmingham NHS Foundation Trust, Birmingham, UK

**Keywords:** T-cell therapy, Chimeric antigen receptor (CAR), Burden of treatment, Personalised-care

## Abstract

**Background:**

Advanced therapy medicinal products such as Chimeric antigen receptor T-cell therapy offer ground-breaking opportunities for the treatment of various cancers, inherited diseases, and chronic conditions. With development of these novel therapies continuing to increase it’s important to learn from the experiences of patients who were among the first recipients of ATMPs. In this way we can improve the clinical and psychosocial support offered to early patient recipients in the future to support the successful completion of treatments and trials.

**Study design:**

We conducted a qualitative investigation informed by the principles of the key informant technique to capture the experience of some of the first patients to experience CAR-T therapy in the UK. A directed content analysis was used to populate a theoretical framework informed by Burden of Treatment Theory to determine the lessons that can be learnt in supporting their care, support, and ongoing self-management.

**Results:**

A total of five key informants were interviewed. Their experiences were described within the three domains of the burden of treatment framework; (1)* The health care tasks delegated to patients*, Participants described the frequency of follow-up and the resources involved, the esoteric nature of the information provided by clinicians; (2) *Exacerbating factors of the treatment*, which notably included the lack of understanding of the clinical impacts of the treatment in the broader health service, and the lack of a peer network to support patient understanding; (3) Consequences* of the treatment*, in which they described the anxiety induced by the process surrounding their selection for treatment, and the feeling of loneliness and isolation at being amongst the very first recipients.

**Conclusions:**

If ATMPs are to be successfully introduced at the rates forecast, then it is important that the burden placed on early recipients is minimised. We have discovered how they can feel emotionally isolated, clinically vulnerable, and structurally unsupported by a disparate and pressured health service. We recommend that where possible, structured peer support be put in place alongside signposting to additional information that includes the planned pattern of follow-up, and the management of discharged patients would ideally accommodate individual circumstances and preferences to minimize the burden of treatment.

## Introduction

Over the last decade, novel advanced therapy medicinal products (ATMPs) i.e., “biological medicinal products that principally exert a pharmacological, immunological or metabolic action” [1], have been developed to treat various cancers, inherited diseases, and chronic conditions [2, 3]. They offer ground-breaking opportunities for the treatment of disease and injury, and by targeting the underlying biology of the disease rather than the symptoms, can significantly reduce the requirement for chronic care and potentially cure the patient [4]. With the largest cell and gene therapy cluster outside of the USA, the UK has become one of the international leaders in this new technology [5] and ATMP clinical trials are increasing by 50% each year, with forecasts that the number of patients treated by ATMPs will rise from 2,500 in 2021 to 10,000 by 2028 [5, 6].

Being at the vanguard of regenerative therapies is a challenge for a pressured National Health Service (NHS) and the trials it supports [4]. Therefore, to ensure the successful completion of treatments and trials in the next wave of novel ATMPs, it's important to learn from the experiences of patients who were among the very first recipients of ATMPs and improve the clinical and psychosocial support offered [7–11].

**Fig. 1:**
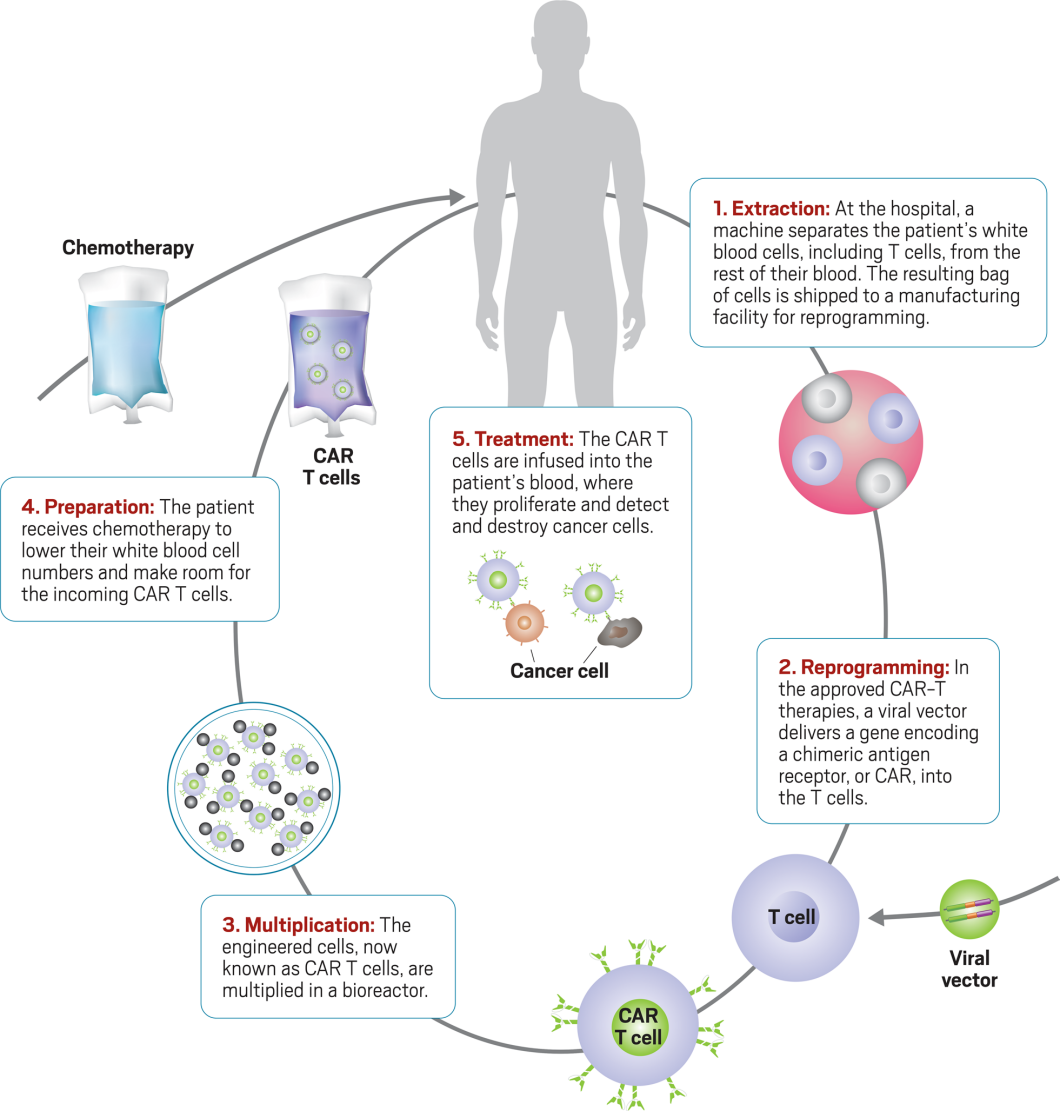
Process of CAR-T-cell therapy [18] Copyright © 2018 by the American Chemical Society and reprinted by permission of the copyright owner

Chimeric antigen receptor (CAR) T cell therapy is a recently introduced ATMP that offers the opportunity to learn from the experiences of some of the first patients and triallists of less well understood ATMPs used in the treatment of cancer. The therapy involves T-cells being harvested from a patient, and then genetically modified to recognise and target a specific protein on the cancer cells, before being reintroduced to the patient where they recognise and attack the cancer cells [12]. The process is summarised in Fig. 1. It is now available on the NHS to treat acute lymphoblastic leukaemia in children and young adults up to 25 years of age, and adult patients with non-hodgkin lymphoma, when two preceding lines of treatment have failed. CAR-T therapy is also being trialled to understand how it performs against other cancers and the duration for which modified T-cells continue to attack the targeted cancer cells [12–15]. Currently, there is limited published work that has explored the experiences of those receiving CAR-T therapy, [16, 17] and none that has explored its introduction in the UK.

The patient experience of any treatment regime is complicated by the finite capacity they possess to attend the work of being a patient whilst meeting the meaningful obligations to family, community, and employers [19, 20]. Discrepancies can emerge between the workload placed on individual patients in pursuing a course of treatment and the resource and capabilities they can draw on in maintaining and managing treatment regimens [21–23]. The requirements placed on patients have been usefully conceptualised as the ‘Burden of Treatment’ by May et al. [11]. It provides a structured means of understanding a patient's perception of their treatment in the context of their individual, dynamic and potentially complex social situations [19, 24, 25].

The work presented here is a qualitative exploration of the burden of treatment placed on the first tranche of CAR-T therapy patients. The findings are presented within the three key domains of the Burden of Treatment framework; the healthcare tasks delegated to f patients; the factors that exacerbate the burden placed on patients; and the consequences of healthcare tasks for patients’ daily lives [26]. As well as informing the care and support of future CAR-T therapy patients, our findings provide practicable insights that enable policymakers, commissioners, trial designers, and care providers to better support those patients receiving novel and less well understood ATMPs.

## Methods

### Study design

We conducted a qualitative investigation informed by the principles of the key informant technique [27] to capture the experience of some of the first patients to receive CAR-T therapy in the UK. The key informant technique is an ethnographic research method based on the precept that not all members of a community will have the necessary experience and perspective to enable understanding of less common circumstances and encounters [28]. In this instance key informants are sought who through their unique position are able to provide the relevant insight and information [28]. The technique has been successfully used to establish patient perspectives in more frequently observed healthcare conditions across a variety of health care systems [29, 30] including colorectal cancer [31] HIV [32] and cardiovascular disease [33]. A directed content analysis [34, 35] was used to populate a theoretical framework informed by Burden of Treatment Theory to determine the lessons that can be learnt in supporting their care, support, and ongoing self-management [7, 26].

#### Ethics approval and consent to participate

Ethical approval was granted by the University of Birmingham’s Science, Technology, Engineering and Mathematics (STEM) ethics committee (ERN_ 21–0196), all methods were performed in accordance with the guidelines and regulations stipulated.

#### Burden of treatment theory

The Burden of Treatment theoretical model acknowledges the impact of the interactions between those that are sick and their employers, social and support networks, and their personal circumstances, as well as the expectations of clinicians, administrators, managers, and policy-makers providing their healthcare [24]. It holds to the premise that (re)designing healthcare services around better coordinated and patient-centred delivery increases the opportunity for patients to successfully negotiate their treatment regimens and self-manage their care [11]. This promotes better experiences of illness, more effective healthcare utilisation, and improved healthcare outcomes [24].

The work presented here utilizes a predefined taxonomy of the Burden of Treatment that used the data from an international survey conducted across 34 countries including the UK to describe and classify the components of the patient workload [26]. We have adapted it to become an analytical framework that delineates the various tasks delegated to patients and the consequences of their compliance with the treatment, including the incidental factors that exacerbate this workload and the impact they have on their daily lives [24, 26]. The framework is summarized in Table 1.

**Table 1:** Burden of treatment framework (after Tran et al.[26])

Domain	Construct	Definition
Healthcare tasks delegated to patients	Administrative tasks	The administrative tasks involved in managing and maintaining the treatment regime. Includes reading the paperwork associated with their healthcare record and treatment schedule
	Condition and treatment follow-up	The responsibility placed on patients to organise the required testing, self-monitoring and planned service utilisation. Includes precautions before/when performing tests and planning, organising and fulfilling visits to the appropriate Health care provider
	Learning and developing an understanding of the illness and treatment	The process of acquiring knowledge of treatment options, side-effects, long term complications, and appropriate self-care [36]. Includes learning how to navigate the healthcare system to access timely and appropriate care associated with the treatment
	Lifestyle changes	The modifiable habits and ways of life that can influence overall health and well-being [37]. Includes changing or maintaining health behaviours to minimise the impact of the treatment such as physical exercise or dietary amendments
	Management of medications	The extent to which a patient takes medication as prescribed, including the correct dose and frequency, and continued, safe use over time [38]. Includes planning and organising medication intake and the safe storage in the home of appropriate amounts of medication
	Organising and performing non-pharmacological treatment	Constituting science-based and non-invasive interventions on human health. intended to prevent, or address health problems [39]. They include products, methods, programs or services such as performing physical therapies
	Organise formal caregiver care	The formal care provided by a healthcare institution or individual (as opposed to the informal care provided by family, or friends) [40]. Includes fulfilling self-monitoring tasks and arranging appointments
Factors that exacerbate the burden of treatment	Nature, time required, and frequency of healthcare tasks	The impact on patients of performing the tasks related to the treatment, its side-effects and the ensuing impact on symptoms. Includes planned and unplanned service utilisation
	Personal factors	Characteristics of individuals includes intrinsic factors relating to resilience and inner resources, and external factors relating to the availability of social support they draw from their family, friends, community, and spiritual beliefs. Alliances built with healthcare providers
	Situational factors	The contextual factors that determine whether and how patients can actively manage their healthcare including changes in personal circumstances (such as a change in job) or in personnel of their healthcare team
	Structural factors	The availability and access of resources directly related to health care services. Includes coordination of care providers and knowledge and training of care providers
Consequences of healthcare tasks on patients’ daily lives	Impact on professional, social, family life, and leisure activities	The professional and social consequences that affect patients due to their treatment This includes balancing the pursuit of meaningful activities with appropriate attention to illness needs and the opportunity cost in professional life
	Financial impact of healthcare tasks on patients	Direct costs of treatment related to the value of resources used in supporting adherence, and indirect costs of treatment such as time lost from work
	Emotional impact	The varying ability of patients to explore, express and process various emotions in response to the loss of health or functioning resulting from the treatment
	Lack of adherence	The extent to which a person's behavior—taking medication, following a diet, and/or executing lifestyle changes—corresponds with the agreed recommendations from a healthcare provider [32]

### Settings/recruitment

At the time referred to in the interviews (2019), CAR-T therapy was only provided at a small number of sites in England, Scotland and Wales. Potential participants i.e., patients in receipt of the first wave of CAR-T therapy were identified through the Midlands and Wales Advanced Therapy Treatment Centre (MW-ATTC). Staff asked those eligible if they could be contacted by the study team and where they agreed they were forwarded a copy of the participant information sheet. They then confirmed their interest and were offered the opportunity to raise any questions before they provided written consent. Each participant was then contacted to arrange a suitable time for an interview via telephone, or video platform dependent upon their preference. Our aim was to recruit up to six individuals in line with recommendations for the key informant technique [41].

### Data collection

Semi-structured interviews were conducted via digital video platform by IL, an experienced qualitative researcher with interests in health service delivery previously unknown to the participants. The interviews explored patient experiences of CAR-T therapy, including perceptions of the information and support provided, the impact and influence of side-effects, and how the requirements of future patients being treated with ATMPs might be met. A summary of the content of the topic guide can be found in Box 1. All interviews were recorded using a digital voice recorder and were transcribed verbatim by a registered transcription service.

**Box 1:** Summary of topic guide

Background information:
Age, time since diagnosis
What did you understand of CAR-T therapy and the treatment process?
What information was provided by care staff?
Did you access information independently? Where from?
What conversations did you have about its suitability and potential use?
Were side-effects discussed?
What was the decision-making process like?
What was your experience of the treatment process?
Who was involved, where?
Were there any side-effects?
What were their impact?
What were you told of your progress?
What were your experiences subsequent to receiving the treatment?
Level of service utilisation (follow-up, unplanned admissions etc.)
Side-effects
What support was available?
Where from? (NHS or others?)

### Analysis

Coding was applied to transcripts by IL using a directed content analysis [34, 35]. This methodology uses existing theory to develop the initial coding framework before beginning the analysis process [42]. In this instance we populated a framework created of the domains and constructs that constitute the Burden of Treatment [26] (see Table 1). Adopting this approach allows for the development and inclusion of any emergent themes or specific examples to be included within the established framework [34, 35]. The data were placed within the relevant domain (and construct) of the framework. Any queries and discrepancies in the coding framework were discussed and consensually agreed with the research team.

## Results

A total of five key informants were identified and approached. All agreed to participate with interviews lasting between 55 and 74 min. We analysed the transcripts for data relating to the three domains and associated constructs of the Burden of Treatment framework [26]. Within each domain a number of examples emerged that related specifically to participants’ experience of receiving CAR-T therapy. These are further explored within the relevant domains and constructs below, alongside illustrative quotes attributed to the pseudonymised participants which includes their participant code, gender and time since diagnosis of the condition for which they were treated.

### Healthcare tasks delegated to patients

Within this domain participants described the impact of several tasks on their ability to complete the treatment. Specifically, these related to the constructs; condition and treatment follow-up, learning and understanding of the treatment and illness, the management of associated medications, and the organisation of formal health care.

#### Condition and treatment follow-up

As with any ATMP, patients are required to attend a number of follow-up appointments after receiving the therapy. There are a number of factors that can influence the degree of testing, monitoring and follow-up of patients that have undergone CAR-T therapy [43] including compliance with the mandated follow-up by the European Society for Blood and Marrow Transplantation and pharmaceutical regulations that stipulate the collection of follow-up data extending to several years. However, in all cases the follow-up involved regular visits to the clinic that gradually reduced in frequency over time [44]. For one participant, the frequency of follow-up visits became an issue due to the distance from the secondary care facility hosting the follow-up appointments. In this instance they explained how the time in transit was often longer in duration than the appointment.

“So, when I did the actual advanced therapy trial… the check-ups which only required me to be there for like half an hour because it was - they'll take some blood, fill in a form and weigh me - was essentially all it was, so that's 20 minutes, half an hour. I had an hour and a half travelling time to get there, and then an hour and a half travelling time to get back, so that was a much more substantial time cost.” P3- Male, 6yrs

However, patients felt the endpoint of the data collection or clinic attendance was undefined. This led to uncertainty in some who were frustrated that they did not know precisely what the endpoint of the therapy might look like and as a result how long the follow-up might continue for:

“…if you turn round and said, ‘You’re 60% fit and the best we can get you is to 70%’, at least that’s an outcome. At the moment, I don’t know. I doubt if I can be 100% but if I knew early doors that…’ look, we hope you’re going to be 100%, but it could be X, Y, or Z…’ then ‘This is the way you can manage your life…by [you] taking these actions and the hospital taking these actions’…to say, ‘That is the end of the journey…’ to a certain extent, ‘…where you’re at.’ - but I feel that it’s open-ended and it hasn’t been completed, if you see what I mean?” P4- Male, 3yrs

Issues with acquiring the necessary understanding of the treatment are explored in further detail in the next section.

#### Learning and developing an understanding of the illness and treatment

Participants described a variety of sources of information they utilised in understanding the treatment, its side-effects, and long-term aftercare. They also spoke about key areas of interest where additional information was sought.

Participants describe how they initially learnt about the treatment from clinicians when they began discussing the option of undertaking CAR-T therapy. These early conversations involved a rudimentary description of what the therapy entailed and how it might impact the patient. However, because of the history of these participants there were no other therapeutic options available to extend their survival beyond six months.

“I asked the consultant, ‘Well what if I decide not to have this particular treatment? That was the first one, first treatment. He said, ‘Well you might see Christmas’ so, you know that was from February to the December. So, but after that, I didn’t really bother asking … I felt that they were there to get you better whatever.” P2-Female, 6yrs

One participant noted that the language used by clinicians was often esoteric and so the participant independently sought additional information from a range of alternative sources, including the internet:

“[I thought] … that language is ‘your language’. It's the language you use with your colleagues to describe ideas and share information and it - and they are the default words you go to. So, it's perfectly reasonable to expect that when you write a document explaining what your outcomes are for a particular thing, you'd use that language because they mean things, and so I tend to find myself - and maybe this is just because of the type of person I am - but I tend to find myself googling a lot of words, going 'What exactly are they referring to here?' and 'What does this mean?' P3- Male, 6yrs

Discrepancies emerged between the information being provided about the material risks of the treatment (in-line with the requirements of gaining informed consent to treat) and what patients wanted to know. One participant described how staff were unable to explain what the entire treatment pathway might look like and that creating some guidance of what to potentially expect over the coming months would be useful to help manage their expectations:

*“*It’s like, ‘[after] two years he has to have a PET scan to just ensure that he’s still 100%.’ and after two years, ‘This is exactly how we’re going to manage him moving forward’…If it could be steered a bit better, then the patient will feel a lot better about themselves...” P4- Male, 3yrs

In another example, a participant was keen to learn more about the experiences of previous patients and the side-effects they experienced, questions that they felt were not appropriate to ask a senior clinician. As they explained:

“…you know, it was like trivial things - well it might sound trivial, but I shouldn’t say that, because its personal isn’t it? - like “Am I going to lose my hair again?” and stuff like that because you know it’s a big trauma to go through. And to have to go through it like three times, I just wanted to know if I needed to prepare myself and all sorts of things like that, like side effects, I just wanted to speak to someone - and there was no one.” P1-FEMALE, 4YRS

There are a range of side effects associated with CAR-T therapy yet certainly at the time of the treatment much was still to be understood about the precise impact on individuals [45]. This meant that for some participants the side-effects they experienced felt uncorroborated, such as bone ache or ‘brain fog’. As two participants described:

“…like difficulties with concentration of word finding, like even now, but I just put that down to how much treatment I've actually had to have and the fact that I'm in medically induced menopause, but you know…” P1-Female, 4yrs

“…Mine was - I was lying in bed - ‘These beds at [name of hospital] are terrible! My hips are killing me!’ It wasn’t. It was the effect of the CAR-T affecting my bone structure” P4- Male, 3 Yrs

#### Management of medications

The experience and perceptions of the medication regimes following treatment varied in accordance with the individual’s clinical history. For those participants where CAR-T therapy had significantly improved their lived experience the impact of their medication was minimal. As one participant explained:

“I must say, I had forgotten how well, well feels. So, that’s really good… I'm still having to take a certain amount of medication as a prophylactic doses. But other than that, you know life is as good as it can be!” P2-Female, 6yrs

However, another participant with a continuing and significant comorbidity continued to feel fatigue which meant that collecting their prescription was a major undertaking:

“My life, at the moment, is very restricted in terms of how much work, if any, I can do during the day, how much I sleep, how much I see my friends. So, the idea that I'm going into town to get my prescription? That to me is a major outing.” P3- Male, 6yrs

#### Organise formal caregiver care

With rare conditions or treatments it is recognised that providers of usual care working outside the team delivering the treatment are unlikely to understand its’ effects on participants, nor how they should respond to participant concerns [46]. One participant explained how this led them to creating their own treatment pathway with their local GP:

*“*I’m pleuritic in the lung because of the dead cell structure, so I’m coughing up a lot of phlegm…I’m thinking...’ Right, now is this pleuritic?... Is it a bit of dead cell stuff coming up? I’m coughing up phlegm, oh, but no B cells. Have I got a chest infection?’ and it was sort of, from my experience, managing it myself. Phone up the doctor. ‘Look, I think that’s a chest infection’. They’re great now in [local town] because I suppose I’m seen as some bigger fish in a smaller pond. ‘Yes, [name of participant], we’ll get some amoxicillin out to your home.’ You know, so that’s quite good as far as that relationship, but I very much manage it myself…” P4- Male, 3yrs

### Factors that exacerbate the burden of treatment

Participants described the factors that exacerbated the burden of the treatment within four constructs; the nature and frequency of healthcare tasks they had to undertake, and a range of personal, situational, and structural factors.

#### Nature, time required, and frequency of healthcare tasks

At follow-up appointments participants were required to provide a blood sample to monitor the physiological impact of the treatment as well as complete a number of questionnaires describing their symptoms or otherwise relating to their well-being and quality of life.

“I got the big fact sheet explaining what they were collecting and why, but after that, the questionnaires were just part of the - 'This is, this is what we need you to do today in, in the check-up,' I never minded doing them. They were nice and easy to do.” P3- Male, 6yrs

However, because it was not clear as to what the questionnaires were designed to elicit, or how the information might be used one participant queried the relevance of some of the content.

“You do feel as if some of the questions are a bit, well almost irrelevant to what you are going through, like ‘Are you happy with your sex life?’ Well, you know is that anything to do with CAR-T therapy? I don’t know?...So, whether or not they're just trying to get a whole well-being type of picture, I don’t know.” P1-Female, 4yrs

In terms of the management of their side effects the impact of CAR-T therapy was relatively minimal, because it was administered to participants who had previously experienced lengthy and often invasive therapies with more significant and wide-ranging side-effects. As one participant explained:

“In comparison with the BMT [bone marrow transplant], which I had maybe like six or seven months before I had CAR-T, it was really a walk in the park, and I felt that myself when I went through it.” P5- Female, 3yrs

#### Personal factors

Personal factors relating to individual beliefs and attitudes and the support drawn from their social context impact on the ability of individuals to manage the patient workload. One participant described how they were reminded of their fortitude by a friend:

“I've got a broad spectrum of friends, church and that sort of thing … so, I remember saying to one person, who reminded me of this, after I became well, and he said, ‘…remember saying to me “as long as I can survive until there's something that can cure me” ‘. P2-Female, 6yrs

Another participant drew solace from being in a position to help others by sharing their experiences on social media.

“I've got a handful of people now who have approached me through social media, sent me a message and said, I've seen your, like posts or someone’s told me about you like, am I okay to ask you a couple of questions about CAR-T? And it’s the same questions that I had. It’s so nice to be in a position now where I can do that for other people.” P1-Female, 4yrs

Where there was direct peer contact (albeit fortuitous) early in the process, it offered immediate benefits and reassurance to the participant about to undertake CAR-T therapy. As one participant described:

“I was saying, ‘Well I'm going on this CAR-T therapy, I don’t really know much about it, but you know that’s what's going to happen to me’. And ___ came over and she said, ‘I hope you don’t mind me butting in, but…’ she said, ‘… I had that!’ and of course she looked so well…You know - that was fantastic!” P2-Female, 6yrs

#### Situational factors

Beyond the consideration of side-effects related to the treatment a participant was concerned about the impact of taking the COVID vaccine considering the depleted nature of their immune system.

“I had my second COVID jab. Now, this is, as I say, where like there’s still grey areas. … ‘Oh, hang on a minute. If I do have the COVID jab, have I got antibodies to fight off COVID?’ So, well, we’re waiting to do the tests or whatever… I’m thinking ‘My T cells are sat there doing such a good job, if this cancer comes, they’re going to zap it out, so I’ve still got my new T cells in there, so my old D cells are rubbish.’… So, I’m worried saying, ‘Well, you’re putting this Pfizer stuff in there. It’s going to affect my T cells. Is it going to, you know, disrupt the cancer thing or whatever? So, there was all that,” P4- Male, 3yrs

#### Structural factors

These were some of the very first patients in the UK to undergo CAR-T therapy and as result there was a lack of understanding of the impact of the treatment in the health service beyond their specialist care team. This meant that sometimes inappropriate advice or recommendations could be provided by other clinicians. As one participant experienced after developing flu-like symptoms:

“I remember when I had a bit of a cold and a temperature about three or four months after CAR-T. I went to A&E, and no one knew what to do with me. I was literally on the phone, the registrar put me on the phone to another registrar - this was at night as well - and she was like, ‘I’ll come and see you at nine o’clock in the morning, you need to wait.’ and I was like, ‘I'm going home mate! You can tell them to take this cannula out of my hand right now and I’ll come home, and I’ll sleep in my own bed…because you're telling me there's no bed for me? That is a disgrace!’ P1-Female, 4yrs

In another example, it took a while for clinicians to understand that the bone ache some experienced following the therapy could be treated by steroids. As one participant explained:

“Rheumatology saw me as a project because they hadn’t come across somebody with CAR-T therapy before. So, the consultant was, listening and listening about these things. He tried everything and he went, ‘[Participant Name], you haven’t got arthritis, we’ve checked you for everything’. I was the first patient CAR-T they’d used the steroid treatment on to alleviate the bone ache. It was a first for them as well.” P4- Male, 3yrs

Because of the uncertainty of trialing such a new treatment, and the seriousness of the consequences if the treatment failed, participants described how they were offered psychological support. One participant was linked with a network of volunteers:

*“*In [hospital name], there are dedicated volunteers that will talk to you if you’ve got a specific concern, or they will take you into a quiet place if you were upset or something like that. So, the department as such tries to look after you.” P2-Female, 6yrs

In another example a participant described how they had benefitted enormously from a formal referral to a trained psychologist:

“[consultant] … he probably looked at me and thought I’m going through a bit of a tough time. I wasn’t crying or anything, but he said, ‘We’ve got a consultant you can go and see, psychology consultant’ I said, ‘Yeah, okay, fine.’ but my thought process was, ‘Why am I bothering…? ‘Cause this is a waste of time!’ Anyway, I’ve gone with [Dr’s name], sat in. Best hour I ever had! She could absorb everything so I could get everything off my chest, and I came out refreshed - more positivity.“ P4- Male, 3yrs

### Consequences of healthcare tasks on patients’ daily lives

Within this domain participants described the impact of the treatment on their social and professional lives, and the emotional consequences of being one of very few to receive CAR-T therapy, including the toll of waiting to hear if they were eligible for treatment.

#### Impact on professional, social, family life, and leisure activities

Participants are expected to make a variety of lifestyle changes in relation to the ongoing effects of the original condition, its symptoms or the side-effects of the treatment, such as a compromised immune response [12]. For example, participants described the pressure of avoiding infection:

“With CAR-T, obviously it destroys all your B cells so, you're missing that big part of your immune system and so I'm on tenterhooks of like literally one infection and they're going to get me on the IV Ig [immunoglobulin].” P1-Female, 4yrs

This vulnerability to infection led participants to considering the degree of contact with others with their concern exacerbated by the onset of the pandemic.

“Wouldn’t it be mad, for the NHS to spend so much on me, for me to walk out the door, pick up COVID and die tomorrow sort of thing, that’s the thing! [they said] ‘Look, your situation is that your IV Ig and IgG levels are still zero. So, from a B cell perspective, your Pfizer jabs aren’t working… you can only go a day a week and be with three people with windows open.’ or whatever...” P4- Male, 3yrs

#### Emotional impact

Participants described the anxiety of awaiting the decision as to whether they would be eligible patients for the treatment aware of the lack of viable alternatives:

“You know, it was tough because I was waiting for [the national panel] to make a decision on my life, basically. So that was, waiting for that phone call on the [Date 2019] was, was, was tough and to be honest, I sort of… I don’t know. I’d started to lose it a bit, that I didn’t think it was going to happen, that I started to sort of,, break down a little bit, I think. It was that time I shut down; you know?” P4- Male, 3yrs

Being one of the first to have such a rare treatment led to a feeling of loneliness for one participant:

“I think at that time, I was the only one in the UK that managed to secure a place. There were others obviously world-wide, but I was the only in the UK. So, I felt a bit lonely, being the only one…” P2-Female, 6yrs

One participant described how the seriousness of their condition and the failed treatments that preceded CAR-T therapy had led to an appreciation of what was important when confronting their mortality:

“I’ve learned so much about myself through this, is that if I hear anybody say, ‘Money is everything’, they’re so far from the truth. They’ve got it so wrong. Life, this is about time. If you haven’t got time, you can’t spend money and, you know, I was always saying…’I’ve got up to six chemotherapy treatments, that gives me another five months living and then if that doesn’t work I can go into stem cell, I’ve got another six months.’ It might sound silly, but that’s how you think [but] in just two minutes, you go from, ‘I’ve got plenty of time!’ to say ‘Oh.’ …[you hear] ‘I’m sorry to say it hasn’t worked. Stem cell’s not going to work for you’, and all of a sudden, you’re looking over the cliff edge. I mean my partner was there, she was nearly on the floor, so you go right to the edge. I saw sort of what my mortality looked like. I had a vision of it all…” P4- Male, 3yrs

## Discussion

### General findings

The rate of development of ATMPs continues to grow with increasing numbers of clinical trials and participants accessing these revolutionary treatments [6]. Although clinical and technical knowledge is advancing, the challenges faced by those who are the early recipients of some of the more novel treatments, such as CAR-T therapy, are less well-understood. The Burden of Treatment theoretical framework has allowed us to describe the impact of CAR-T therapy treatment and its follow-up, and our findings have identified areas where further support can improve the experiences of subsequent patients that are the first to be treated with novel ATMPs.

In considering the healthcare tasks delegated to them, participants described the frequency of follow-up and the resources involved in travelling to a clinic located within a single location; the esoteric nature of the information provided by clinicians and how the lack of alternatives precluded more detailed conversation around the impact of CAR-T therapy. Many sought additional information from the internet or social media and lamented more informal support they might consult over side-effects they feared clinicians may consider superficial. The burden of their treatment was exacerbated by a number of factors, these included confusion around the relevance and purpose of questionnaires completed during follow-up consultations, and the lack of understanding of the clinical impacts of the treatment in the broader health service, compounded by the absence of a network of peers that may have offered emotional and experience-based support. Finally, in considering the consequences of the healthcare tasks and treatment, patients described the emotional impact of being amongst the first to receive the treatment, namely the anxiety induced by the process surrounding their selection for treatment, and the feeling of loneliness and isolation engendered by being one of the first patients. Below we describe the findings within each domain in more detail, placing them in the context of existing research.

### Specific findings

#### Healthcare tasks delegated to patients

The difficulties posed by patients travelling to specialist clinics hosted by large secondary care facilities in major conurbations is not confined to patients undergoing CAR-T therapy and have been seen in some other ATMP treatments such as gene therapies [47–49]. This burden of travel is known to aggravate anxiety and depression in several patient groups [50] and support with travel costs has been proposed within the NHS's New National Framework for Non-emergency Patient Transport [51]. However, it is a particularly important consideration for those patients with rare conditions or undergoing novel treatments because of how the additional severity and complexity impacts their ability to arrange and undertake travel [52].

Reducing the time spent on travel can help minimise the impact and disruption to patients with rare diseases and treatments in the UK [53] and the NHS has considered moving some specialty clinics into community settings [54, 55]. There have also been recommendations for more equitable availability and greater utilisation of telemedicine for those patients requiring specialist care [49]. However, at the time of the study many ATMPs and certainly CAR-T therapy could only be delivered in Europe within Joint Accreditation Committee of the International Society for Cellular Therapy and EBMT (JACIE) accredited centres [56]. Meanwhile, examples of more fundamentally patient centric care pathways for those receiving ATMPs are beginning to emerge in the United States where the delivery of CAR -T therapy is being piloted in outpatient clinics [57] or otherwise via more holistic care pathways designed by multiple disciplines which alongside specialist clinicians, include social workers, psychologists, and dieticians [58–60].

Our participants described the widely recognised issue of clinicians reliance on technical and medicalised language when discussing treatment options [61]. That additional information and clarification was independently sought suggests that increased effort should be devoted to meeting ATMP patient requests for further education [16]. This includes content on the general concepts of drug development and risks specific to ATMPs [62–64]. The recent introduction of specialist CAR-T therapy nurses is now helping alleviate the issue by providing a more accessible yet reliable source of information [65].

Limited guidance as to what follow-up and aftercare might consist of was described, and the importance of managing the expectations of other ATMP patients regards their aftercare has been recognized previously [63, 64]. In other settings protocols have emerged for discussing serious illnesses and intensive treatment regimens with patients, where their content addresses the pattern of follow-up, including the frequency of visits, the length of time they are in the system, and the types of tests and scans they can expect to undertake [66–68].

#### Factors that exacerbate the burden of treatment

Previous research has indicated that patients are more likely to be accepting of the demands placed on them by their treatment (as well as the harms, risks and constraints of their medication) if they can place it in the context of the benefit they expect to receive [69]. Our participants had previously undertaken at least two alternative treatments, some of which had extensive and severe side effects, such as bone marrow transplants [70]. This and the finality of their position meant they were initially better placed to accept the impact of the tasks associated with CAR-T therapy. They also understood that receiving a novel treatment meant they would be closely monitored as clinicians attempted to gain as much information as possible on its impact [71], a process which included the completion of multiple questionnaires. The value of such patient-completed questionnaires in assessing the safety, efficacy and tolerability of treatment is widely recognised in many patient groups including patients with rare diseases [72] and particularly where formalised as patient-reported outcomes [73–76]. However, participants described a lack of clarity as to the aim of some of the questionnaires and there are limits to patient compliance with this type of paperwork [77]. One way in which engagement can be maintained is by ensuring patients remain aware of what the questionnaires are designed to capture and how that data will be used [78].

As with some other ATMPs [79, 80], much of the published research on patient experience of CAR-T therapy has focused on the exploration of side-effects and its impact on symptoms [17, 81] or the ethical issues of interrupting access to beneficial treatments [82]. However there are important considerations of the management of the transition of patient participants to usual care in community settings [83]. Less is known of the interactions between patients and the broader health system subsequent to their treatment, and certainly not of patients that are amongst the first to receive a new treatment [84]. In the UK, issues of communication between primary and secondary care settings have long been recognised [85], and are further complicated by the short-term provision of CAR-T care via tertiary or quaternary services [86]. These issues are particularly apparent when patients with rare conditions present in primary care [87] or emergency departments [88]. Empowering patients offers one solution [89] but not all patients are comfortable assuming a more active role [90] and improving lines of communication between specialists and colleagues involved in managing complex patients remains a pressing issue [91] though is not intractable for all ATMPs as demonstrated by the role of primary care in gene therapies in the United States [92]. Financial incentives and improved e-messaging have been suggested [93] as has the issuing of ‘cards’ to patients such as those used to avert adrenal crises [94].

#### Consequences of healthcare tasks on patients’ daily lives

Patients described the considerable emotional impact of waiting on third parties to decide their eligibility for CAR-T therapy and their ‘loneliness’ in being one of very few in their position, with concerns about their long-term safety shared by other ATMP patients such as those undergoing gene therapy [95]. At the time they were treated only some 30 individuals in the UK had undertaken CAR-T therapy. Although some emotional and psychological support was made available via volunteers or trained practitioners there was no readily available peer support leading one patient to contact others via social media and establish their own network of peers. The succor gained from talking to peers reflects a growing body of evidence indicating the benefit of such support in a range of patients, including those with rare diseases and [96–98].

### Strengths and limitations

The Burden of Treatment framework we used to analyse the data has proven a useful tool in identifying and understanding the various elements that contribute to the ‘workload' of patients that are amongst the first to receive a novel and less well understood ATMP, in this instance CAR-T therapy. It was not intended to be a definitive identification of every issue faced by the first patients to pass through a trial or receive the treatment of a less common ATMP, but it successfully allowed us to surface the types of issues that might arise so they may be further explored and addressed. We acknowledge that the work might be usefully expanded by exploring the experiences of patients that have been the first to experience similarly new ATMPs. In that sense this is the beginning of a conversation about how the very first patients to experience a treatment are managed and cared for.

By definition only a small number of patients can be amongst the first to receive a treatment and the number of key informants is within previously adopted levels [41]. Our participants also met the recommended characteristics of key informants in that they are knowledgeable, communicative, and their unique position (in being amongst the first patients to receive CAR-T therapy) allows for significant insight into the phenomenon [99, 100], and a perspective we could not otherwise obtain [101].

## Conclusions

If ground-breaking ATMPs such as CAR-T therapy are to be successfully introduced at the rates forecast then it is important that the experiences of early recipients are shared and lessons learnt to minimise the anxiety, and disruption for the individual involved but also to provide maximal learning for ensuing iterations of the technology. We have described how these early patients can feel emotionally isolated, clinically vulnerable, and structurally unsupported by a disparate and pressured health service. Structured peer support, access to additional information including the likely pattern of follow-up, and the consideration of patients’ individual circumstances and preferences for care would all contribute to reducing the overall burden of treatment.

## Data Availability

The datasets generated and/or analysed during the current study are not publicly available due to limitations of ethical approval involving the patient data and anonymity but are available from the corresponding author on reasonable request.
